# Theta-burst stimulation entrains frequency-specific oscillatory responses

**DOI:** 10.1016/j.brs.2021.08.014

**Published:** 2021-08-21

**Authors:** Ethan A. Solomon, Michael R. Sperling, Ashwini D. Sharan, Paul A. Wanda, Deborah F. Levy, Anastasia Lyalenko, Isaac Pedisich, Daniel S. Rizzuto, Michael J. Kahana

**Affiliations:** a University of Pennsylvania, Perelman School of Medicine, Philadelphia PA 19104, USA; b University of Pennsylvania, Department of Psychology, Philadelphia PA 19104, USA; c Thomas Jefferson University Hospital, Department of Neurology, Philadelphia PA 19107, USA; d Thomas Jefferson University Hospital, Department of Neurosurgery, Philadelphia PA 19107, USA; e Nia Therapeutics Inc., Bala Cynwyd, PA 19004, USA

**Keywords:** Theta burst stimulation, Oscillations, LFP, Intracranial EEG, Functional connectivity

## Abstract

**Background:**

Brain stimulation has emerged as a powerful tool in human neuroscience, becoming integral to next-generation psychiatric and neurologic therapeutics. Theta-burst stimulation (TBS), in which electrical pulses are delivered in rhythmic bouts of 3–8 Hz, seeks to recapitulate neural activity seen endogenously during cognitive tasks. A growing literature suggests that TBS can be used to alter or enhance cognitive processes, but little is known about how these stimulation events influence underlying neural activity.

**Objective:**

Our study sought to investigate the effect of direct electrical TBS on mesoscale neural activity in humans by asking (1) whether TBS evokes persistent theta oscillations in cortical areas, (2) whether these oscillations occur at the stimulated frequency, and (3) whether stimulation events propagate in a manner consistent with underlying functional and structural brain architecture.

**Methods:**

We recruited 20 neurosurgical epilepsy patients with indwelling electrodes and delivered direct cortical TBS at varying locations and frequencies. Simultaneous iEEG was recorded from non-stimulated electrodes and analyzed to understand how TBS influences mesoscale neural activity.

**Results:**

We found that TBS rapidly evoked theta rhythms in widespread brain regions, preferentially at the stimulation frequency, and that these oscillations persisted for hundreds of milliseconds post stimulation offset. Furthermore, the functional connectivity between recording and stimulation sites predicted the strength of theta response, suggesting that underlying brain architecture guides the flow of stimulation through the brain.

**Conclusions:**

By demonstrating that cortical TBS induces frequency-specific oscillatory responses, our results suggest this technology can be used to directly and predictably influence the activity of cognitively-relevant brain networks.

## 1. Introduction

Theta-burst brain stimulation has been used in research and clinical settings since the 1980s [1], including recent use in humans via non-invasive methods such as transcranial magnetic stimulation (TMS). By delivering rhythmic bouts – or “bursts” – of high-frequency stimulation, 3–8 times per second, theta-burst stimulation (TBS) mimics patterns of endogenous 3–8 Hz theta oscillations seen in local field potentials [2,3]. Because TBS recapitulates natural brain rhythms, neuroscientists have hypothesized that it may be efficacious for modulating the activity of brain circuits responsible for cognitive processing or underlying disease [4].

Recent work using non-invasive forms of TBS, including TMS and transcranial direct current stimulation (TDCS), suggests that TBS can improve symptoms of depression [5–7], and enhance working [8,9] and episodic memory [10–12]. Invasive TBS via surgically-implanted electrodes – though still an emerging technology and significantly less common – has also been shown to improve episodic memory [13]. Moreover, prior work in animals has shown that TBS can induce synaptic long-term potentiation (LTP) [14,15] or depression (LTD) [16], depending on the pattern and duration of stimulation. This work was paralleled in humans in a theta-burst TMS paradigm targeted to motor cortex, demonstrating facilitation or inhibition of synaptic transmission, depending on the pattern of stimulation [17]. Collectively, these experiments indicate that TBS induces long-lasting change in brain function and physiology.

There is mounting evidence that TBS renders change to the brain, but the way in which TBS directly influences neural signals is unknown. Human studies of invasive non-TBS stimulation paints a mixed picture: some evidence suggests neural responses will generally align with the stimulation frequency [18,19], while others have found that low-frequency responses can be readily observed even following high-frequency (>100 Hz) stimulation [20,21]. However, there is broader agreement that evoked responses to cortical stimulation are constrained by functional and structural architecture [20,22–25]. Noninvasively, several studies have used functional magnetic resonance imaging (fMRI) as a lens to examine TBS-induced changes in BOLD signal and connectivity, while others assessed changes in cortical oscillations using scalp electroencephalography (EEG). In both cases, investigators have found evidence that TMS-TBS can alter BOLD-based functional connectivity in targeted networks [7,10] and modulate cortical oscillatory power, particularly in the theta band [9,26]. However, these studies typically focus on long-term changes in neural signals induced by repeated TBS pulse trains, reporting on induced effects after minutes or days. Though an important piece of the TBS puzzle, there are also immediate neural responses to brief stimulation events that go unexplored in these paradigms. Understanding such real-time changes in neural dynamics – particularly at the level of intracranial electrophysiology – is crucial if TBS is to be applied in closed-loop paradigms for cognitive enhancement or therapeutic effect [13,27,28]. Moreover, the brain’s response to exogenous perturbations could shed light on the fundamental nature of oscillatory local field potentials, an area of intense study but with limited explanatory models.

Indeed, perhaps the most straightforward question about the neural influence of TBS – does a volley of TBS induce theta oscillations in the brain? – remains a largely unanswered question. One recent study examined whether deep brain TBS of the basal ganglia yielded immediate change in cortical oscillations, finding that dorsolateral prefrontal (DLPFC) theta power increased following less than 30 s of intermittent theta-burst stimulation [29]. However, this study does not offer a full accounting of how intracranial TBS may influence neural activity across a range of recording locations and stimulation frequencies. And if TBS does provoke oscillatory responses, important aspects of their physiology are unknown, such as whether neural tissue can be entrained to an artificial frequency or whether the brain can maintain a provoked oscillation in the absence of external inputs.

Here we use intracranial cortical TBS to answer key questions about how the brain responds to exogenous electrical inputs: (1) does TBS induce theta oscillations in the brain, and are the oscillatory responses specific to the stimulation frequency, (2) how rapidly do induced rhythms arise, and for how long do these rhythms persist after stimulation offset, and (3) are oscillations induced in regions functionally connected to the stimulation site? To this end, we recruited 20 patients with indwelling electrodes undergoing presurgical planning for refractory epilepsy. By delivering direct cortical TBS across a range of stimulation targets and stimulation frequencies, we were able to deeply characterize the response of neural tissue to a stimulation event, finding that theta oscillations can be – but are not always – evoked in regions both local and remote to the stimulation site. Additionally, the theta response to stimulation is immediate, robust, and specific to the stimulated frequency, typically lasting for several hundred milliseconds after stimulation is discontinued. Finally, underlying brain architecture – as measured by structural factors and functional connectivity – is significantly but not exclusively predictive of how stimulation events propagate through the brain.

## 2. Methods

### 2.1. Participants

Twenty patients with medication-resistant epilepsy underwent a surgical procedure to implant subdural platinum recording contacts on the cortical surface and within brain parenchyma. Contacts were placed so as to best localize epileptic regions. Data reported were collected at 6 hospitals over 4 years (2015–2018): Thomas Jefferson University Hospital (Philadelphia, PA), University of Texas Southwestern Medical Center (Dallas, TX), Dartmouth-Hitchcock Medical Center (Lebanon, NH), Hospital of the University of Pennsylvania (Philadelphia, PA), Mayo Clinic (Rochester, MN), and the National Institutes of Health (Bethesda, MD). Prior to data collection, our research protocol was approved by the Institutional Review Board at participating hospitals, and informed consent was obtained from each participant.

### 2.2. Electrocorticographic recordings

iEEG signal was recorded using grid, strip, and stereotactic depth electrodes (contacts spaced 3.5–10 mm apart) using recording systems at each clinical site. iEEG systems included DeltaMed XlTek (Natus), Grass Telefactor, and Nihon-Kohden EEG systems. Signals were sampled at 500, 1000, or 1600 Hz, depending on hardware restrictions and considerations of clinical application. Signals recorded at individual electrodes were first referenced to a common contact placed intracranially, on the scalp, or mastoid process. To eliminate potentially confounding large-scale artifacts and noise on the reference channel, we next re-referenced the data using a bipolar montage. The resulting bipolar timeseries was treated as a virtual electrode and used in all subsequent analysis. As determined by an expert neurologist, channels exhibiting highly non-physiologic signal due to damage or misplacement (4.2% of total), or with significant ictal discharges (16.6%), or those placed within the seizure onset zone (3.4%), were excluded from all analyses.

**Fig. 1.**
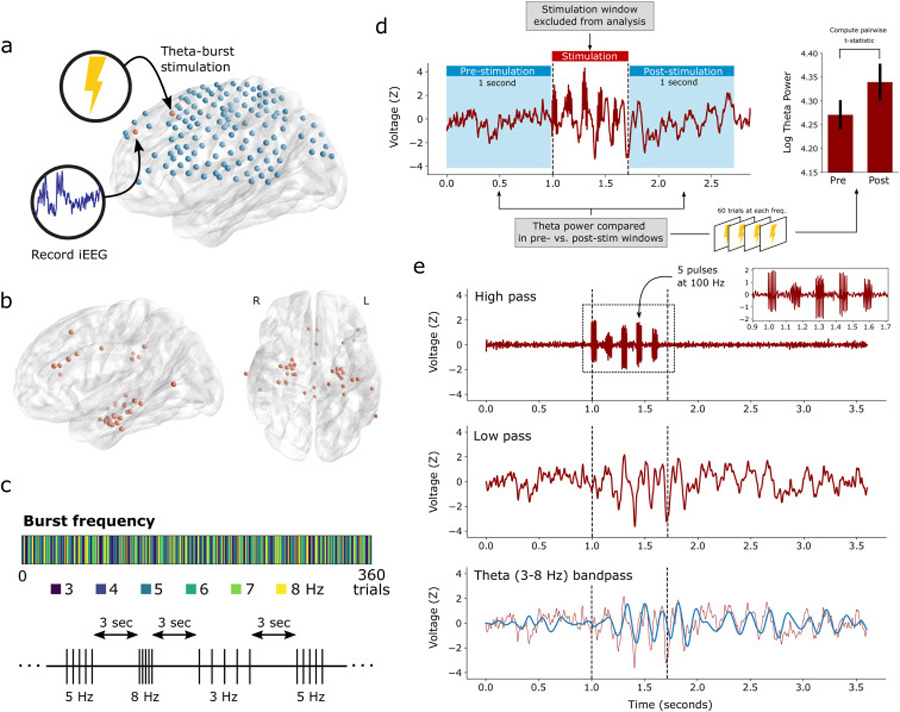
Experimental paradigm and analysis procedure. (**a**) Trials of theta-burst stimulation (TBS) were delivered at a specific target site during each experimental session, while continuous iEEG was recorded from all other electrodes. (**b**) 39 unique sites were stimulated across 20 subjects. Targets were not randomly selected, with sites in the medial temporal lobe (MTL) preferentially picked for use in related studies. Non-MTL sites include lateral temporal cortex, cingulate gyrus, angular gyrus, and dorsolateral prefrontal cortex. (**c**) 360 stimulation trials were delivered for each experimental session, with varying burst frequencies (3–8 Hz) randomly interleaved. Trials were spaced by 3 s, with ± 200 ms of random jitter. (**d**) Example trial from a recording site in the left DLPFC. Theta spectral power was measured in 1-s windows before and after stimulation events using the multitaper method. To avoid the influence of stimulation artifact, we excluded stimulation-period activity from our analyses. Across 60 trials at each stimulation frequency, the difference in theta power between pre- and post-stimulation windows were compared with a paired *t*-test. Error bars indicate ± 1 SEM over trials. (**e**) Visual representation of signal components. Stimulation artifact appears as high-frequency fluctuations reflecting the 100–200 Hz pulses. Underlying low-frequency rhythms are evoked by the stimulation event and persist after stimulation offset.

Unlike in studies of a related dataset [20,21], the TBS paradigm used in this study does not generate a significant post-stimulation artifact, except for the stimulated electrodes themselves. Therefore, we did not adopt an explicit artifact-rejection algorithm in the post-stimulation interval. However, we note that stimulated electrodes were always excluded from all analyses, and all primary analyses were carried out with no contamination from the during-stimulation interval (Fig. 1; see “Spectral power analysis” for details). Raw electrophysiogical data and analysis code used in this study is freely available at http://memory.psych.upenn.edu/Electrophysiological_Data.

### 2.3. Anatomical localization

Anatomical localization of electrode placement was accomplished using independent processing pipelines for depth and surface electrode localization. For patients with MTL depth electrodes, hippocampal subfields and MTL cortices were automatically labeled in a pre-implant, T2-weighted MRI using the automatic segmentation of hippocampal subfields (ASHS) multi-atlas segmentation method [30]. Post-implant CT images were coregistered with presurgical T1 and T2 weighted structural scans with Advanced Normalization Tools [31]. MTL depth electrodes that were visible on CT scans were localized within MTL subregions by neuroradiologists with expertise in MTL anatomy. Subdural electrodes were localized by reconstructing whole-brain cortical surfaces from pre-implant T1-weighted MRIs using Freesurfer [32]. Regions of interest (ROI) were identified by the Desikan-Killiany cortical parcellation after mapping final contact locations to individual Freesurfer space.

### 2.4. Stimulation paradigm

All stimulation was performed with Blackrock Cerestim and Blackrock splitter box and cabling. At the start of each session, we determined the safe amplitude for stimulation using a mapping procedure in which stimulation was applied at 0.5 mA, while a neurologist monitored for afterdischarges. This procedure was repeated, incrementing the amplitude in steps of 0.5 mA, up to a maximum of 2.0 mA for depth electrodes and 3.0 mA for surface electrodes (chosen to be below the afterdischarge threshold and below accepted safety limits for charge density [33]). For each stimulation session, we passed electrical current through a single pair of adjacent electrode contacts (“bipolar” stimulation). Stimulation was delivered using charge-balanced biphasic rectangular pulses (pulse width = 300 μs), in 5 bursts of 5 pulses delivered at 100 or 200 Hz (see Fig. 1). Over trials, burst frequency was varied between 3 Hz and 8 Hz by randomly selecting the inter-burst interval from 333 ms, 250 ms, 200 ms, 167 ms, 143 ms, or 125 ms, until 60 trials at each theta frequency had been delivered (360 total trials per session). Pulse amplitude was held fixed for each stimulation site according to the predetermined safe threshold, ranging from 0.25 to 2.0 mA for depth electrodes and 0.5–2.5 mA for surface electrodes. Trials were spaced by 3 s, with up to ± 200 ms of randomly-applied jitter added to the interval. During a session, subjects were instructed to sit quietly and did not perform any task. An average of 1.95 stimulation sites were selected for each subject, spanning sites within the MTL and distributed cortical regions (see Fig. 1b). Putative seizure onset zones (SOZ) were avoided for purposes of experimental stimulation; 4/9 subjects with identified and reported SOZs were medial temporal lobe, while the remainder had onset zones spanning prefrontal, insula, lateral temporal, and parietal cortices.

### 2.5. Spectral power analysis

**Fig. 2.**
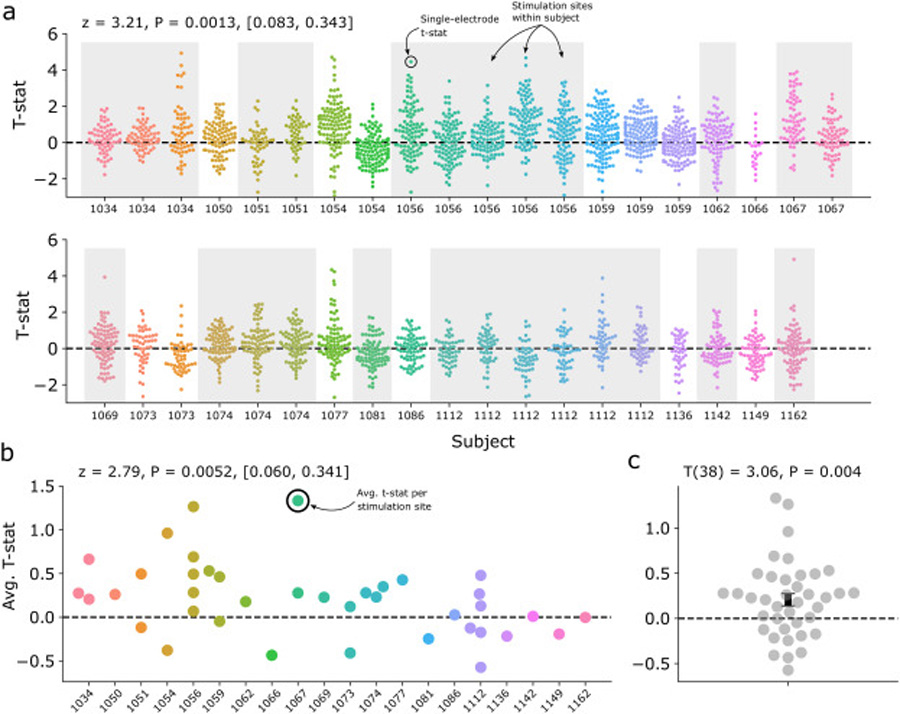
TBS evokes increases in post-stimulation theta power. (**a**) A pre-vs. post-stimulation T-statistic is computed for each recording electrode in each experimental session, reflecting the degree to which stimulation evoked increases (or decreases) in post-stimulation theta power. The distribution of T-statistics across electrodes is plotted for each experimental session, with subjects delineated by gray or white boxes. Using a hierarchical linear mixed-effects model, there is a significant positive effect of stimulation at the population level (*z* = 3.21, *P* = 0.0013, Intercept: [0.083, 0.343] 95% CI), indicating that TBS significantly increased post-stimulation theta power in our sample. The finding is consistent when only modeling effects at the session-level (*z* = 2.79, *P* = 0.0052, Intercept: [0.060, 0.341] 95% CI) (**b**) or without using a hierarchical approach and treating sessions as independent measures (1-sample *t*-test, *t* (38) = 3.06, *P* = 0.004) (**c**). Colors are for visual differentiation only.

**Fig. 3.**
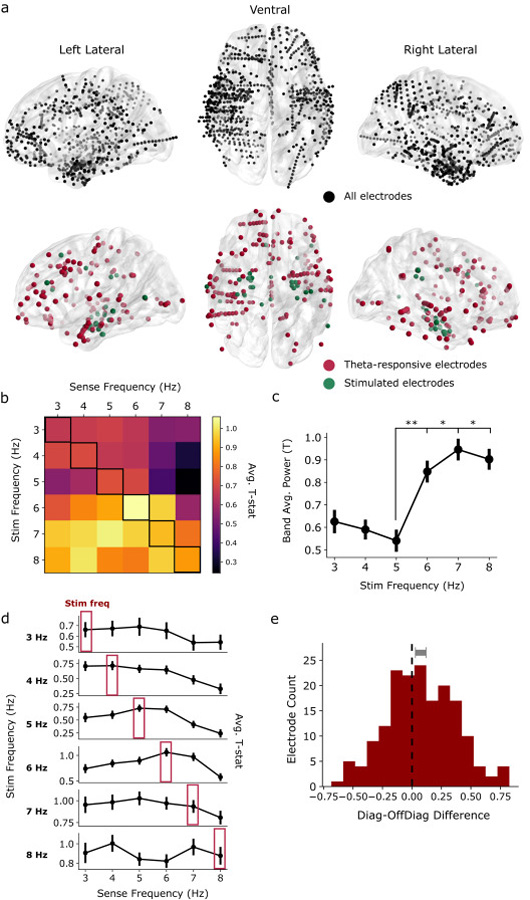
Theta-responsive electrodes exhibit frequency-specific oscillations. (**a**) *Top*: placement of all 1534 electrodes in the dataset. *Bottom*: Location of the 174 electrodes that exhibit a significant theta response (red) and the 29 corresponding stimulation locations (green). (**b**) Matrix depicting the mean theta-frequency response (“sense frequency”; columns) to each stimulation frequency (rows). Warmer colors indicate a stronger oscillatory response. (**c**) Band-averaged theta response to each stimulation frequency. Higher stimulation frequencies tend to evoke greater post-stimulation theta power. SEMs over electrodes are depicted for visualization purposes, but pairwise statistics are calculated using a hierarchical LMM approach (*p < 0.05, **p < 0.01; see Methods). (**d**) Frequency-specific power response (average *t*-statistic) to each stimulation frequency. Red boxes indicate the stimulation frequency analyzed, which often corresponds to the maximal power response frequency (i.e. 4 Hz, 5 Hz, 6 Hz). Error bars show ± 1 SEM over electrodes. (**e**) Frequency-specificity was quantified by taking the difference between the average of diagonal and off-diagonal elements in the stimulation/sense matrix, for each electrode. Assessed at the electrode-level by an LMM, the average *t*-statistic along the diagonal of the matrix (i.e. the frequency-specific response) is significantly greater than the average value of the off-diagonal elements (Wald test, *z* = 2.23, *p* = 0.026). Gray bar shows 95% CI of the mean.

We used the multitaper method to assess spectral power in the pre- and post-stimulation intervals (–1000 ms to stimulation onset, and +1000 ms after stimulation offset; Fig. 1d). We avoided the Morlet wavelet method to obviate the need for buffer periods that extend into the stimulation window. All spectral analyses were performed with the MNE Python software package [34]. Signals for 3–8 Hz theta power analyses were downsampled to 50 Hz; signals for analyses at higher frequency bands were downsampled to 100 Hz or 500 Hz, ensuring adequate sampling relative to the measured frequency. For each trial, we measured the PSD from 3 to 8 Hz in 1 Hz increments, using a time-bandwidth product of 3 and excluding tapers with <90% spectral concentration. To compute a *t*-statistic for each electrode (Fig. 2), we first log-transformed the power values and then averaged over theta frequencies. The pre-vs. post values were compared with a paired *t*-test, which were then used in a hierarchical linear mixed effects model (see “Statistical approach”). For the frequency-specific analysis presented in Fig. 3, *t*-statistics were computed similarly, at each frequency within the 1 Hz band. In spectral analyses at higher frequencies (9–13 Hz alpha, 16–28 Hz beta, 30–50 Hz gamma and 75–200 Hz HFB; Supplemental Fig. 2), we excluded the 100 ms immediately preceding and following each stimulation trial, to avoid any possibility that high-frequency pulse artifact would contaminate our results. Additionally, to account for the possibility that a small number of electrodes or trials may exhibit corruption of the post-stimulation signal, we excluded any electrode from analysis if the theta power *t*-statistic was greater than 5.

**Fig. 4.**
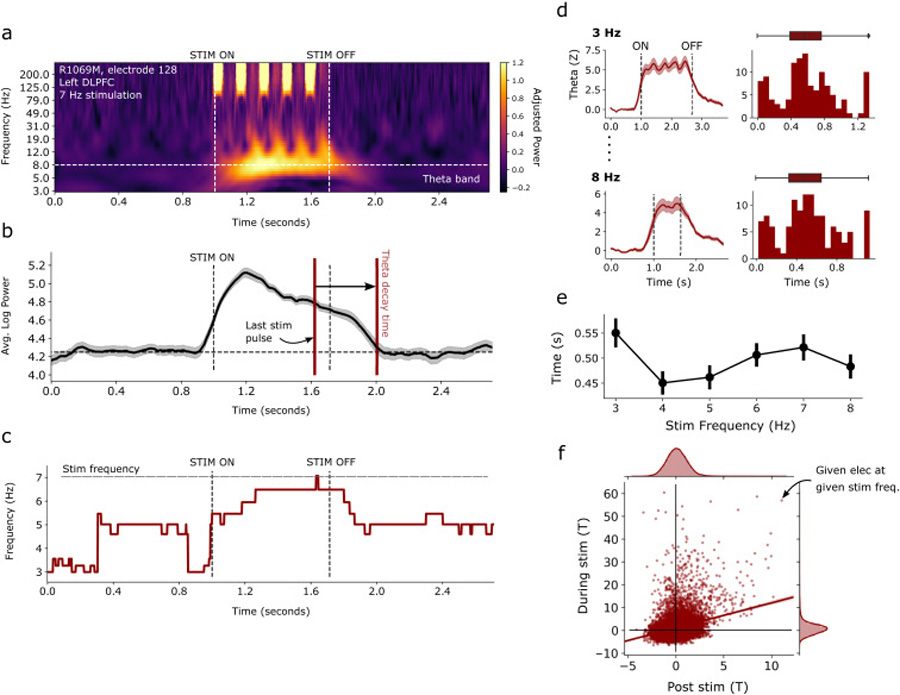
Onset and persistence of induced theta responses. (**a**) Time-frequency spectrogram of an example electrode in the DLPFC, averaged across all 7 Hz stimulation trials and normalized against the pre-stimulation baseline. A ~7 Hz response in the theta band can be seen, alongside high-frequency spectral artifact present above 100 Hz. (**b**) In the example electrode, spectral power was averaged in the 3–8 Hz band to generate a time-varying measure of theta power. Theta power tends to peak early in the stimulation interval and decays gradually. Note that theta power begins to ascend slightly prior to stimulation onset due to convolutional effects. The “theta decay time” is taken as the duration between the last stimulation pulse and theta power decaying to within 1 standard deviation of the baseline. Error bars show ± 1 SEM over trials. (**c**) In the example electrode, frequency of maximum spectral power as a function of time. Note that 7 Hz stimulation drives a near-7 Hz response, which decays to a baseline rhythm after stimulation offset. (**d**) *Left:* Average theta timecourses for all response electrodes, shown for 3 Hz and 8 Hz stimulation frequencies. Other frequencies are qualitatively similar. *Right*: Distribution of theta decay times across all response electrodes. (**e**) Mean theta decay time across electrodes as a function of stimulation frequency. Average decay time falls between 0.45 and 0.55 s for all stimulation frequencies. Error bars show ± 1 SEM over electrodes. (**f**) Correlation between post-stimulation and during-stimulation power across all electrodes and stimulation frequencies in the dataset (Pearson *r* = 0.31).

To construct the time-frequency measures of theta power over each trial (Fig. 4), we convolved the EEG signal from each electrode with taper windows 2 cycles in length (MNE function “tfr_multitaper”), to minimize temporal windowing which could contaminate pre- and post-stimulation intervals with artifactual signal. We also used time-bandwidth products of 2, to achieve greater frequency specificity for the analysis presented in Fig. 4d and e. Next, we (1) averaged the time-frequency response in the frequency domain from 3 to 8 Hz, (2) averaged over trials, and (3) *z*-scored relative to the 0–500 ms prestimulation baseline, ultimately generating a theta timecourse for each electrode. The “theta decay time” was taken as the difference between the time of last stimulation pulse and the time the trial-averaged signal fell to within 1 standard deviation of the 0–500 ms prestimulation baseline. Decay times were computed separately for trials corresponding to each stimulation frequency (Fig. 4d and e). Electrodes for which theta power never exceeded a z-score of 2.0 were not included in this analysis.

The during-stimulation *t* values used in Fig. 4f were computed similarly to those in Fig. 2, instead comparing matched pre-vs. during-stimulation taper windows according to the duration of stimulation in a given trial. *T* values corresponding to during-stimulation and post-stimulation power were extracted for each electrode at each stimulation frequency (N_elecs_ × N_freq_ values), and correlated for each subject/session using Pearson correlation. We adopted a permutation test for significance to correct for the non-independence of t-statistics across electrodes and stimulation frequencies. To do this, we measured the Pearson correlation for every possible 1-shift of each vector against the other, and again for the mirror image of that vector. This procedure resulted in a distribution of chance correlations that maintained statistical dependencies, against which we compared the true correlation to obtain a *P*-value.

### 2.6. Identification of theta-responsive electrodes

To focus on the subset of electrodes that exhibited a significant theta response to stimulation, we adopted a simple procedure to identify theta-responsive electrodes in each subject, for each stimulation session. To do this, we considered any electrode with a theta *t*-statistic greater than 2.0 as exhibiting a significant response. This procedure yielded 174/1534 (11.3%) significant electrodes across 29/39 (74.3%) experimental sessions and 16/20 subjects (80%). Similarly, to assess for the presence of electrodes with stimulation-induced decreases in theta power (Fig. 6a), we took all electrodes with a theta *t*-statistic less than −2.0. This thresholding procedure was not done for the purpose of statistical inference – rather, it was used to identify a subset of electrodes for further characterization. As such, we adopted a statistical threshold that does not correct for multiple comparisons.

### 2.7. Functional connectivity estimation

We obtained coherence values between electrode pairs using the MNE Python software package. The coherence (*C*_*xy*_) between two signals is the normalized cross-spectral density (Equation (1)); this can be thought of as the consistency of phase differences between signals at two electrodes, weighted by the correlated change in spectral power at both sites.

(1) $$C_{xy}=\left|\frac{S_{xy}}{S_{xx}S_{yy}}\right|$$

where *S*_*xy*_ is the cross-spectral density between signals at electrodes *x* and *y*; *S*_*xx*_ and *S*_*yy*_ are the auto-spectral densities at each electrode. Consistent with prior studies of EEG coherence [20], we used the multitaper method to estimate spectral density. We used a time-bandwidth product of 4 and a maximum of 8 tapers (tapers with spectral energy <90% were removed), computing coherence for frequencies between 5 and 13 Hz. Inter-electrode coherences were computed for a series of 1-s windows extracted sequentially from 10-s baseline periods of a non-stimulation task, in which subjects wait passively before beginning a verbal free-recall task. Subjects typically had 24–72 such baseline periods, but all had a minimum of 10 periods (i.e. the minimum total number of windows used for network estimation was 100). To construct the low-frequency networks, cross-spectra were first averaged across all baseline period windows, normalized by the average power spectra, and then averaged between 5 and 13 Hz.

**Fig. 5.**
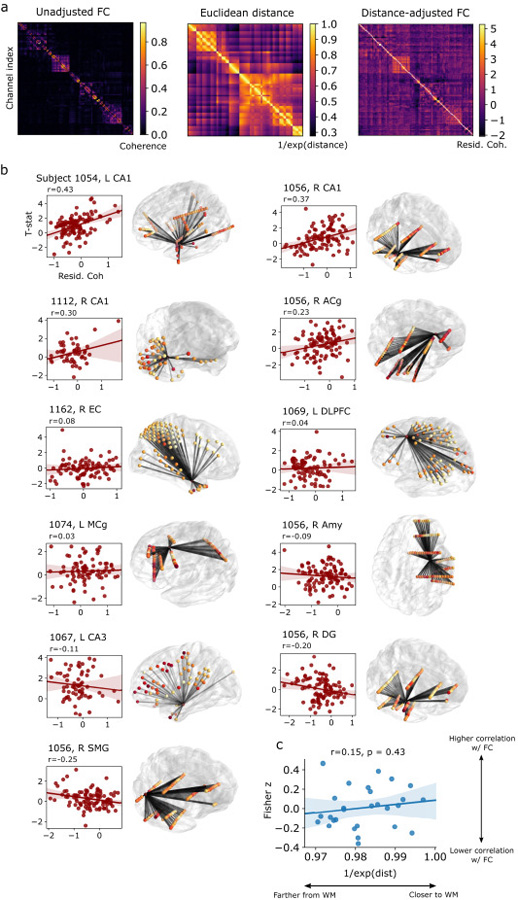
Power-connectivity correlations. (**a**) *Left:* Extraction of functional networks in an example subject. Functional networks are first measured using 5–13 Hz spectral coherence during a baseline period. The adjacency matrix representation reflects coherence between all possible pairs of electrodes in the subject. *Middle*: Inter-electrode distances in the same subject (after a normalizing transformation; see Methods). *Right:* Residual coherence after regressing out distance. Distance-adjusted values are used to assess whether stimulation-induced theta power is correlated with functional connectivity. (**b**) Residualized coherence was correlated against theta power *t*-statistics for every recording electrode, using a hierarchical LMM. There was a significant effect of connectivity among subjects with at least two theta-responsive electrodes (LRT, χ [2](4)=15.8, *p* = 0.003, β = 0.165). Representative scatter plots and Pearson correlation coefficients are shown for several stimulation site used in the model, in order to visually convey the underlying data and variability between stimulation sites. Stimulation sites and subject identifiers are listed above each plot. Brain renderings depict higher *t*-statistics as redder colors, and stronger functional connections as darker lines. (**c**) The (Fisher z-transformed) correlation coefficients for all 28 stimulation sites were correlated against each target’s proximity to the nearest white matter tract (normalized distance, 1/*e*^distance^). No significant relationship was found (*r* = 0.15, *p* = 0.43). ACg: anterior cingulate; EC: entorhinal cortex; DLPFC: dorsolateral prefrontal cortex; MCg: midcingulate; Amy: amygdala; DG: dentate gyrus; SMG: supramarginal gyrus.

To account for the brain’s tendency to densely connect nearby regions, and the possibility of volume-conducted effects of stimulation, we regressed coherence on inter-electrode distance, and used the (distance-independent) residualized coherence values in our subsequent analyses. To do this, we first measured the Euclidean distance between all possible pairs of electrodes, and normalized the values by taking the reciprocal of their exponential (i.e. a Euclidean distance of 0 could correspond to 1.0). We normalized all inter-electrode coherence values by taking the logit transform. Next, we used linear regression to correlate distance and coherences, and used the resulting model find the residual, distance-independent coherence. This procedure essentially reduces strong coherences between nearby electrodes, but may emphasize the coherence between distant electrodes. See Fig. 5a for a representative example. Subsequently, we used an LMM to estimate the relationship between induced theta power and residualized connectivity across all electrodes, analyzing the subset of subjects with at least one theta-responsive electrode, or two theta-responsive electrodes, or the entire dataset (see “Statistical approach” for further details).

To analyze the relationship between connectivity effects and white matter proximity (Fig. 5c), we first computed the distance from each electrode to the nearest white matter. Distance were measured as the normalized Euclidean distance from a stimulation electrode (i.e. bipolar midpoint of the anode/cathode) to the nearest vertex of that subject’s Freesurfer white matter segmentation [35] based on T1 MRI. Next, for each subject, we computed the Pearson correlation coefficient (*r*) between electrode *t*-statistics and the functional connectivity (residualized coherence) linking each recording electrode to the stimulation electrode (i.e. scatter plots in Fig. 5b). This resulted in a single *r* value for each stimulation site, reflecting the degree to which functional connectivity predicts downstream theta responses. Finally, these *r* values were Fisher-z transformed and correlated with the distance of each stimulation site to white matter (Fig. 5c).

**Fig. 6.**
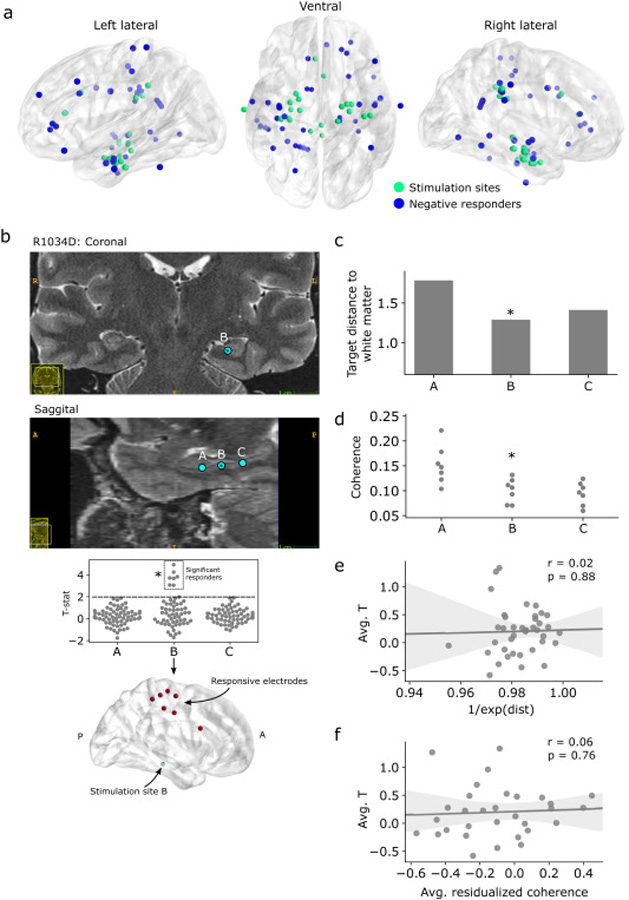
Characterizing between-subject variability in response to TBS. (**A**) Location of the 40 electrodes with a decrease in post-stimulation theta power (T < −2; blue) and corresponding 22 stimulation locations (green). (**B**) *Top:* T2-weighted MRI of subject R1034D depicting the placement of three stimulation targets within the left dentate gyrus (blue circles). The only site which induced a significant theta response is labeled “B” and denoted with an asterisk throughout the figure. *Bottom*: Distributions of theta *t*-statistics reflecting stimulation-induced theta power at each site. (**C**) Distance of each stimulation target in R1034D to the nearest white matter voxel. (**D**) 5–13 Hz coherence between each target site and the R1034D’s theta-responsive electrodes. (**E**) Correlation between (normalized) distance to white matter and average stimulation-induced theta power across all electrodes, for each subject/target. Proximity to white matter did not correlate with induction of greater theta power (*r* = 0.02, *P* = 0.88). (**F**) To assess whether functional connectivity predicts between-subject variability in response to TBS, we computed the average functional connectivity of each stimulation site, by taking the mean across all (distance-residualized) coherences to a site. This statistic is the “node strength” in graph theoretic terms. There was no significant correlation between node strength and brainwide response to TBS for each subject/target (*r* = 0.06, *P* = 0.76).

To quantify the overall level of functional connectivity between a stimulation site and the rest of the brain (Fig. 6f), we computed the average of a target site’s coherence to all other electrodes. This statistic is also referred to as “node strength” in graph theory, reflecting the general connectedness of a node to the rest of the network. This statistic was correlated with the average induced theta power for each stimulation site, calculated by averaging the pre-vs. post-stimulation *t*-statistic across all electrodes in a given subject.

### 2.8. Statistical approach

Due to the possibility of within-subject correlations across stimulation sites and recording electrodes, and in consideration of the variable number of stimulation sites and electrodes between subjects, we adopted a hierarchical linear mixed effects modeling (LMM) approach to major statistical analyses in this manuscript. LMMs can account for variability as a function of random and fixed effects, with random effects generally referring to variables of which only a subset from the population have been measured (e.g. subjects, stimulation sites). In this manuscript, we used the LMM implementation in the statsmodels Python package [36]. To analyze the general, brain-wide response to stimulation (Fig. 2 and Supplemental Fig. 2), we used an intercept-only LMM to model the variability of electrode *t*-statistics across stimulation locations (N = 39) and subjects (N = 20), specifying sessions within subjects as random effects. We next used the Wald test to assess significance of the intercept term, asking whether *t*-statistics significantly differed from zero in our population. This test generates a *z* (sometimes called *t*) value by dividing the estimated model coefficient by its standard deviation. We used an identical approach to assess the significance of the frequency-specific response as depicted in Fig. 3c.

To measure the effect of stimulation frequency on band-averaged theta power, we included stimulation frequency as a categorical fixed-effect in our model (Fig. 3b), and used subsequent pairwise Wald tests to assess for significant differences between stimulation frequencies. Finally, we included distance-residualized coherence (see “Functional connectivity estimation”) as a fixed effect to quantify the relationship between functional connectivity and induced power (Fig. 5). To assess the significance of this variable, we used the likelihood ratio test (LRT), in which the likelihood ratio between two nested models (i.e. a full model versus a reduced model absent the fixed effect) follows a chi-square distribution. In both cases, we allowed for random intercepts and random slopes with respect to the fixed effect in question.

## 3. Results

We sought to characterize the moment-to-moment influence of intracranial TBS on neural signals, with a particular focus on induced activity in the theta range. To that end, we delivered intermittent theta-burst stimulation events (“trials”) via indwelling electrodes in 20 neurosurgical patients (Fig. 1a and b). Each trial consisted of 5 bursts of 100–200 Hz biphasic, bipolar stimulation with inter-burst intervals corresponding to 3, 4, 5, 6, 7 or 8 Hz stimulation (see Methods for details). Each experimental session consisted of 360 trials with stimulation frequency randomly interleaved (Fig. 1c). Only one bipolar pair was used for stimulation in each stimulation session, with an average of 1.95 sessions/targets per participant (mode = 1 session).

Because stimulation tends to corrupt iEEG traces with nonphysiologic artifact, our approach in this manuscript was to (1) avoid directly analyzing signal while stimulation was being actively delivered and (2) avoid analysis methods which could introduce artifact from the stimulation period to non-stimulated intervals. For all non-stimulated electrodes, we first clipped 1-s segments of iEEG from the pre- and post-stimulation intervals, and then used the multitaper method to extract spectral power from these signals (Fig. 1d and Methods). Thus, in most analyses, we analyze “pure” iEEG signal with no possible contamination from stimulation artifact (see “Evolution of induced theta power over time” for an exception).

### 3.1. TBS increases post-stimulation theta power

We first asked whether TBS induces theta oscillations at non-stimulated electrodes in the brain. To answer this, we used paired *t*-tests to assess the degree of pre-vs. post-stimulation change in theta band power (3–8 Hz) across all trials, for each electrode in the dataset (Fig. 1d and e). Resulting positive *t* statistics reflect stimulation-related increases in theta power. Fig. 2 depicts the distribution of *t* statistics for each subject/experimental session, at the level of individual electrodes (Fig. 2a) and averaged into session-level effects (Fig. 2b and c). To account for the differing numbers of electrodes and sessions for each subject – and the possibility of within-subject correlations across electrodes and sessions – we used hierarchical linear mixed-effects models (LMMs) to understand the effect of stimulation on theta power. Modeling at the electrode level, with sessions nested within subjects as a random effect, we found a significant positive effect of stimulation (Wald test, *z* = 3.21, *P* = 0.0013, Intercept: [0.083, 0.343] 95% CI), and we note that this finding holds true modeling only session-level variability (Wald test, *z* = 2.79, *P* = 0.0052, Intercept: [0.060, 0.341] 95% CI) or by simply treating sessions as independent observations (1-sample *t*-test, *t* (38) = 3.06, *P* = 0.004). This indicates that TBS stimulation, at the population level, significantly increases theta power in the post-stimulation interval.

### 3.2. TBS induces frequency-specific theta oscillations

Having established that TBS generally increases theta power in non-stimulated brain regions, we next sought to understand whether induced theta rhythms in these areas were tied to the stimulation frequency. Because many electrodes exhibited no significant response to stimulation (Fig. 2b), we first identified the subset of electrodes which were driven by TBS in general, regardless of the specific stimulation frequency in the theta range. To do this, we selected all electrodes with a theta power *t*-statistic greater than 2.0 (this threshold is used solely to identify a subset of electrodes for characterization of theta responses, and not for purposes of direct statistical inference). This selection yielded 174/1534 (11.3%) stimulation-responsive electrodes across 29/39 (74.3%) experimental sessions and 16/20 subjects (80%). Unless stated otherwise, all subsequent analyses were performed on this “theta-responsive” subset of electrodes and subjects.

The 174 electrodes that exhibited a theta-band response were widely distributed among recorded brain regions and resulted from stimulation across a variety of targets, including intra- and extra-MTL sites (Fig. 3a and Supplemental Table 1). Specifically, we identified responding electrodes within the medial temporal lobe, lateral temporal cortex, frontal lobe, and parietal lobe – regions that overlap with the most densely-sampled brain regions in our neurosurgical cohort. Among these electrodes, the general band-averaged theta response was greater with higher-frequency stimulation (Fig. 3b and c). However, by analyzing the theta response at specific frequencies within the 3–8 Hz range, we found that TBS at a specific frequency was more likely to induce power at that same frequency (LMM Wald test, *z* = 2.23, *p* = 0.026, Intercept: [0.008, 0.120] 95% CI; Fig. 3c). Stimulation frequency and maximal theta response frequency were the same at 4, 5, and 6 Hz. Though this effect was statistically significant, a substantial theta response could also be observed at off-target frequencies (see off-diagonal elements of Fig. 3c and d), suggesting that stimulation may entrain a specific frequency which could drift over time, or provoke endogenous mechanisms to generate theta rhythms at a preferred natural frequency. Although we found no evidence for frequency specificity at 7 and 8 Hz stimulation, stimulation at these frequencies appeared to induce strong theta responses across the entire band, suggesting a possible ceiling effect.

### 3.3. Evolution of induced theta power over time

Our earlier analyses indicate that theta-burst stimulation induces frequency-specific rhythms across diverse cortical areas in 1-sec intervals after the offset of stimulation. But when relative to stimulation onset do these rhythms begin, and for how long afterwards do they last? Answering these questions necessitates an examination of the timecourse of stimulation-related theta modulation, including the stimulation interval itself – a period we previously avoided to mitigate the effect of possible stimulation artifact. Because stimulation pulses are delivered at 100–200 Hz, artifact is likely to principally affect the high-frequency range, though we cannot rule out the presence of low-frequency spectral distortion. The ensuring analyses examine the stimulation and peri-stimulation intervals and evaluate the hypothesis that stimulation-period theta activity reflects physiologic versus nonphysiologic processes.

We performed a time-frequency analysis to assess the onset and persistence of induced theta power after stimulation offset. In order to generate a continuous measure of time-varying theta power, we used convolutional windows 2 cycles in length, so as to minimize potential contamination from stimulation artifact in the post-stimulation interval (see Methods for details). As outlined in Fig. 4, we constructed time-frequency spectrograms for each trial, averaged the resultant power in the theta band, and then z-scored relative to the pre-stimulation baseline, generating a normalized measure of time-varying theta power (Fig. 4a–c; see Supplementary Fig. 1 for additional example spectrograms). We then calculated the time between stimulation offset (i.e. the last stimulation pulse) and the moment theta power fell to within 1 standard deviation of the baseline, called the “theta decay time.”

Across all theta response electrodes and stimulation frequencies, theta power increases markedly upon initiation of stimulation, and remains stable until stimulation offset (Fig. 4a–d). Subsequently, we found an average theta decay time of 0.50 s, meaning that substantial theta power persists for approximately half a second after the last stimulation pulse is delivered, with little meaningful effect of stimulation frequency (Fig. 4e). Noting also that the theta power signal is highly asymmetric around the stimulation interval (Fig. 4d), we consider it unlikely that the persistence of theta power post-stimulation is simply an artifact of spectral convolution (but this does account for the apparent rise in theta power slightly before the onset of stimulation). Additionally, we found that there was a small proportion of electrodes (approx. 8%) which exhibited theta decay times greater than our prespecified 1 s post-stimulation window (see histograms in Fig. 3d). These long-decay electrodes were not considered when calculating summary statistics of the distributions.

To assess the degree to which theta power during the stimulation interval was affected by artifactual spectral influences, we correlated the during-stimulation power with the ensuing post-stimulation power for each electrode and stimulation frequency. We used non-sliding multitaper windows to extract spectral power from the during-stimulation and post-stimulation intervals and thereby avoid temporal leakage. If power during the stimulation interval were solely driven by hardware artifact and not physiologic responses, we would not expect a significant correlation with post-stimulation power, which is free of hardware influences. Across all 39 unique stimulation sites in the dataset, we found a mean Pearson correlation coefficient of 0.26 ± 0.19 STD (see aggregated data in Fig. 4f), which was statistically significant for 25/39 of the sites (permutation test, *P* < 0.05, see Methods for details). During-stimulation *t*-statistics mainly fell between −5 and +10, whereas post-stimulation statistics tended to fall between −5 and +5. Some unusually high during-stimulation *t*-statistics were observed (e.g. >20), which may reflect artifactual responses. Therefore, while we still cannot rule out the presence of low-frequency artifact, the data suggest that true physiologic theta responses occur during the stimulation interval. Taken together with our earlier findings, we have demonstrated that stimulation induces a strong, rapid-onset oscillatory theta response, which decays relatively quickly – within half a second – once stimulation is discontinued.

### 3.4. Functional connectivity mediates the effect of theta-burst stimulation

Using continuous (non-theta burst) stimulation, Solomon, et al. (2018) reported that the spectral coherence predicted which downstream areas would exhibit greater oscillatory responses [20]. This finding paralleled other studies which used noninvasive functional measures to similarly predict the propagation of stimulation events through the brain [23,37]. Here, we investigated whether the same relationship holds true in our TBS dataset. We first measured baseline functional connectivity networks using low-frequency (5–13 Hz) coherence, the frequency band shown in Solomon et al. (2018) to interact with stimulation effects. (Six subjects were excluded from this analysis because resting-state data was not available, see Methods.) These networks reflect correlated low-frequency activity between all possible pairs of electrodes in a subject (Fig. 5a). Next, we residualized inter-electrode coherences on Euclidean distance, to account for the brain’s tendency to densely connect nearby regions [38] or propagate effects through volume conduction. Distance-corrected measures of coherence were included as a factor in a LMM, essentially correlating the theta *t*-statistic of a recording electrode with the strength of its coherence to a stimulation electrode. The resulting model coefficient indicates, independent of distance, the degree to which 5–13 Hz coherence predicts TBS-induced change in theta power at a recording site.

Among the subset of subjects with at least one significant theta response electrode and viable resting-state data (N = 10, see representative examples in Fig. 5b), we found a positive but nonsignificant correlation between adjusted coherence and downstream theta power (likelihood ratio test [LRT], χ [2](4)=6.73, *p* = 0.15, β = 0.037). Hypothesizing that power-connectivity correlations are noisy among subjects with very few stimulation-responsive electrodes, we reanalyzed the data among the subset of subjects with at least two theta responsive electrodes (N = 7; 13 stimulation sites). In this smaller cohort, we found a significant correlation between connectivity and induced power (LRT, χ [2](4)=15.8, *p* = 0.003, β = 0.165). This indicates that functional connectivity, independent of distance, is predictive of where TBS will instigate theta rhythms elsewhere in the brain. However, we remark that some stimulation sites exhibited no correlation with functional connectivity, while others showed negative correlations (see Fig. 5b). The 3 TBS sites with the highest power-connectivity correlation were in hippocampal CA1, collected from three unique subjects. We obtained similar findings when analyzing all available subjects and sessions (14 subjects, 28 sessions), regardless of whether any significant theta response electrodes were found (LRT, χ [2](4)=12.1, *p* = 0.017, β = 0.067).

A prior study from our group demonstrated that the correlation between evoked power and coherence was itself contingent on the proximity of a stimulation target to white matter [20]. We performed the same analysis here, asking if the power-coherence correlation coefficient (*r*; see examples in Fig. 5b) for each site was higher if stimulation occurred closer to white matter. Among the 28 stimulation sites available for our functional connectivity analysis, we found no reliable effect of white matter proximity (Pearson *r* = 0.15, *p* = 0.43; Fig. 5c). As the range of distances to white matter tracts was poorly sampled (our prior study included stimulation targets placed directly in white matter tracts), and the number of stimulation targets is relatively small, we cannot draw strong conclusions from this null result.

### 3.5. Between-subject, between-target variability in response to TBS

The previous analysis suggests reasons why nearly 80% of the electrodes and 20% of the subjects in our dataset exhibited no significant response to TBS – the stimulated areas may have not had strong functional or structural connections to other regions. However, it is still remarkable that most of the electrodes in our dataset were essentially unresponsive to TBS. If TBS is to be used for therapeutic purposes, characterizing the variability and absence of response to TBS is just as important as understanding the nature of positive responses. To that end, we analyzed the subjects and regions where TBS appeared to induce no significant response, or a notable negative deflection in theta power. Specifically, we sought to understand across-subject variability in response to stimulation by comparing the overall effect of stimulation to structural and functional features of the target site.

We found significant post-stimulation decreases in theta power in fourteen subjects (70%) among 22 stimulation targets (56%), defined by thresholding for *t*-statistics less than −2 (Supplemental Table 2). Each of these stimulation targets was associated with an average of 1.8 negatively responsive electrodes, far fewer than the average of 6 electrodes per positive target. In total, 40/1534 (2.6%) of electrodes were negatively modulated, which is not greater than the number expected by chance under assumptions of independence (binomial test, *P* = 0.23). Negatively responding sites were widely distributed through recorded cortical areas and were not qualitatively associated with stimulation of a particular region (Fig. 6a; see also Supplemental Table 2). These results are consistent with our earlier analysis demonstrating a population-level effect in the positive direction, but do not strictly rule out physiologically meaningful decreases in theta power within specific subjects.

We also noted stimulation sites which evoked minimal or no observable theta power response. Subject R1034D, for example, underwent TBS at three distinct sites within the left dentate gyrus. Only one of those sites induced a significant theta response, observed as increased power in medial frontoparietal cortex and cingulate gyrus (Fig. 6a). The site which induced significant responses was marginally closer to white matter tracts (stimulation region “B”; Fig. 6b), suggesting that sufficient engagement of fibers by electrical stimulation may be necessary to cause robust remote responses. Notably, stimulation region B lacked strong functional connections with the responsive electrodes, measured via resting-state 5–13 Hz coherence (Fig. 6c). Conversely, stimulation region A – which did not induce any significant responses – featured relatively strong coherence with the electrodes that responded to stimulation at region B.

This subject serves as a counterexample to our earlier analysis showing population-level correlations between functional connectivity and induced power. However, extending this analysis to the entire cohort did not reveal any systematic association between the overall degree of induced theta power and site-specific structural factors (i.e. white matter proximity) or functional connectivity (i.e. target site coherence). The proximity of a stimulation target to white matter was uncorrelated the brainwide average *t*-statistic (Fig. 4e), as was a stimulation target’s overall functional connectedness (Fig. 4f; see Methods for details). Taken together, the utility of a stimulation target to maximize theta response is not a simple function of proximity to white matter tracts or a site’s functional connectedness. We later discuss several other factors which may explain the variability of neural responses to TBS (see Discussion).

### 3.6. Induced effects in higher frequency bands

Our primary focus in this study was to characterize the real-time cortical response to TBS in the theta band – the natural rhythms which are most likely to be influenced by theta-patterned stimulation. However, intracranial stimulation is known to influence activity in higher frequency bands, particularly in the gamma (30–50 Hz) and high-frequency broadband (HFB; ~75–200 Hz) ranges [21,39]. Neural activity in these ranges are known to be cognitively-relevant [40] and sometimes interact with ongoing theta oscillations [41]. Indeed, HFB is considered to be a biomarker of local cortical activity and is correlated with the fMRI BOLD signal [42,43]. To that end, we used the same paradigm as described earlier (see Fig. 1 and Methods) to analyze stimulation-induced changes in the gamma and HFB ranges, solely within the post-stimulation interval. We found that, at the population level, TBS did not induce a significant change in gamma power (LMM Wald test, *z* = 0.936, *P* = 0.349, Intercept: [−0.057, 0.162] 95% CI), but there was a significant increase in HFB power (*z* = 3.241, *P* = 0.001, Intercept: [0.065, 0.263] 95% CI; Supplemental Fig. 2a). This suggests that TBS may provoke increases in 75–200 Hz power in the immediate post-stimulation period, a finding that is consistent with the effect of high-frequency continuous stimulation in a different dataset [21].

### 3.7. Long-term changes in cortical properties

TBS experiments classically examine prolonged changes in cortical excitability or functional connectivity induced by patterned stimulation for several minutes or longer [4,10,17], unlike the real-time assessment we used here. While our particular stimulation paradigm is different than typical intermittent TBS (iTBS) or continuous TBS (cTBS) designs, we nonetheless wondered whether we could find evidence of long-term change in cortical activity, beyond the moment-to-moment oscillations we previously analyzed. To do this, we binned each stimulation session (24 min) into quartiles (6 min each) and asked whether the theta power induced by each stimulation event changed over the course of a session. Among the cohort of 16 subjects with theta-responsive electrodes, we did not find a significant effect of time on theta power (repeated-measures ANOVA, *F* = 1.54, *p* = 0.21; Supplemental Fig. 3) or in higher frequency bands (*P* > 0.05). This suggests that stimulation-induced change spectral power is not more or less likely with longer periods of stimulation. However, this analysis does not rule out regional or subject-specific effects that may be obscured in the average.

## 4. Discussion

Brain stimulation will be key to forthcoming neurologic and psychiatric therapeutics, but the excitement is not yet linked to a rigorous understanding of how electrical stimulation influences neural activity. Paradigms which leverage human intracranial stimulation and recording are especially valuable because they enable a careful and spatiotemporally precise assessment of how neural signals respond to stimulation in a clinical population. In this study, we turned our attention to theta-burst stimulation, which has seen growing interest in recent years due to its putative ability to mimic natural brain rhythms and engender synaptic plasticity [14,17,44]. In a cohort of 20 neurosurgical patients, we characterized the physiology of brainwide LFPs induced by short theta-burst events. We confirmed that TBS induces theta rhythms at sites both remote and local to the stimulation target, in-line with a subject’s underlying functional connectivity profile. Theta responses were immediate, robust, and frequency-specific, and persisted for several hundred milliseconds following offset of stimulation. However, some targets were unassociated with downstream changes in theta power, possibly reflecting whether nearby white matter tracts were accessible to the stimulated electrodes.

By painting detailed picture of how the brain responds to TBS, these findings extend a deeper literature that has examined how other types of stimulation influence neural signals in real-time. Notably, we again found low-frequency power increases in the immediate post-stimulation interval, replicating findings from prior human studies that used higher-frequency stimulation designs [20–22]. These similarities raise the possibility that induced low-frequency responses may be common to exogenous stimulation in general, perhaps revealing a “preferred” natural rhythm of brain tissue as it reacts to perturbation. Future work could examine how low-frequency rhythms evoked by high-frequency stimulation differ from the oscillations encountered in this study. Low-frequency power may also – in part – reflect stimulation-evoked potentials, which are well-documented in the literature [25,45,46]. However, our determination that induced responses are specific to the stimulation frequency suggest that genuine theta oscillations are a substantial component of the measured low-frequency power. Moreover, we demonstrated that theta power evoked during stimulation was correlated with theta power post-stimulation, indicating the persistence of an induced oscillation and not solely a low-frequency evoked potential.

We could not offer a full accounting for why some stimulation targets yielded dramatic increases in theta power, while others appear to cause no discernible change in brain activity and yet others caused theta power decreases. Anecdotal evidence from single subjects suggests the proximity of stimulation to white matter may play a role, which intuitively aligns with expectations regarding the propagation of signals through the brain and recent behavioral evidence [47]. However, our population did not show a significant effect of white matter proximity – or functional connectedness – indicating that other factors dictate the efficaciousness of a stimulation site. The cortical layer most strongly engaged by stimulation might be a key factor, though this is difficult to ascertain with macroelectrodes. And while we excluded electrodes placed in areas with noted epileptic pathology, it is possible that disease-related differences in cortical excitability influenced the effect of stimulation between targets. Future work should establish a full set of biological parameters – including disease status – that can be used to predict the effect of stimulation across brain regions.

Considering the potential therapeutic use of TBS, our results indicate that TBS can immediately provoke theta oscillations in the brain, which have profound functional relevance to cognition [3,48]. Theta oscillations are hypothesized to be a substrate for the domain-general formation of associations, helping us to link multimodal sensory inputs, memories, and abstract knowledge into useful constructs [49–51]. As part of that function, theta oscillations serve as an inter-regional phase reference [52] which organizes the spiking activity of neuronal assemblies [53–55]. The use of TBS to provoke theta oscillations raises the possibility that we can selectively intervene to strengthen specific associations [27,56,57], or encourage the reactivation of associational constructs – like episodic memories – when necessary.

However, significant work remains before we can realize the goal of closed-loop cognitive modulation. In addition to better understanding the anatomical factors which determine how the brain responds to TBS, we must also account for variability in the temporal domain. There is mounting evidence that the effects of stimulation are contingent on the endogenous neural activity near the stimulation site and elsewhere in the brain [27,28]. For example, it is possible that volleys of TBS must arrive in-phase with endogenous theta oscillations to exert their maximal effect, or that strong oscillations must not already be present in stimulated tissue. Teasing apart the relationship between exogenous and endogenous activity would be a key advance in both engineering efforts to design neural interfaces and neuroscience more broadly. Unfortunately, the current study design did not include periods of “sham” stimulation, making it difficult to differentiate the interaction between stimulation and endogenous rhythms from the natural tendency for oscillatory power to drift over time.

Finally, we still have a poor understanding of LFP responses to stimulation as it is actively ongoing. In this study, we deliberately adopted a conservative approach to the issue, presenting only a brief examination of the stimulation interval and thereby avoiding the possible influence of nonphysiologic stimulation artifact. However, as suggested in our data, stimulation likely co-occurs with the strongest neural response and demands more detailed study. As stimulation-induced artifact can manifest in subtle and heterogenous ways – affected by recording systems, patient factors, and referencing schemes, among others – it will be an ongoing challenge for investigators to interpret these signals accurately. Some investigators have adopted artifact-removal methods and sought to analyze signals during active intracranial stimulation [18,22,58], which constitutes a viable approach to the problem. Intra-stimulation analyses are even more convincing if they can be tied to other variables that are known to be unaffected by artifact, such as behavioral changes, pre- or post-stimulation functional connectivity, or long-term alterations in cortical properties.

## 5. Conclusions

Brain stimulation as a technique in basic and clinical neuroscience is here to stay, but its effects on neurophysiology are difficult to measure and largely unexplored. Here, leveraging the high spatiotemporal resolution of iEEG, we demonstrated that exogenous theta-burst stimulation could drive robust, frequency-specific oscillatory responses in real-time. These results are a crucial underpinning for ongoing efforts to optimize brain stimulation in the treatment of disease, and as a tool for understanding the basic elements of neural electrophysiology.

## Supplementary Material

SupplMaterial

## Data Availability

Raw electrophysiogical data and analysis code used in this study is freely available at http://memory.psych.upenn.edu/Electrophysiological_Data.
