# Supplementary material for: Theta-burst stimulation entrains frequency-specific oscillatory responses
Source: Brain Stimul. Author manuscript; Available in PMC 2022 Jun 2. (PMC9161680; doi:10.1016/j.brs.2021.08.014)
Supplement: SupplMaterial [file NIHMS1808782-supplement-SupplMaterial.docx]

**Supplemental Figures and Tables**


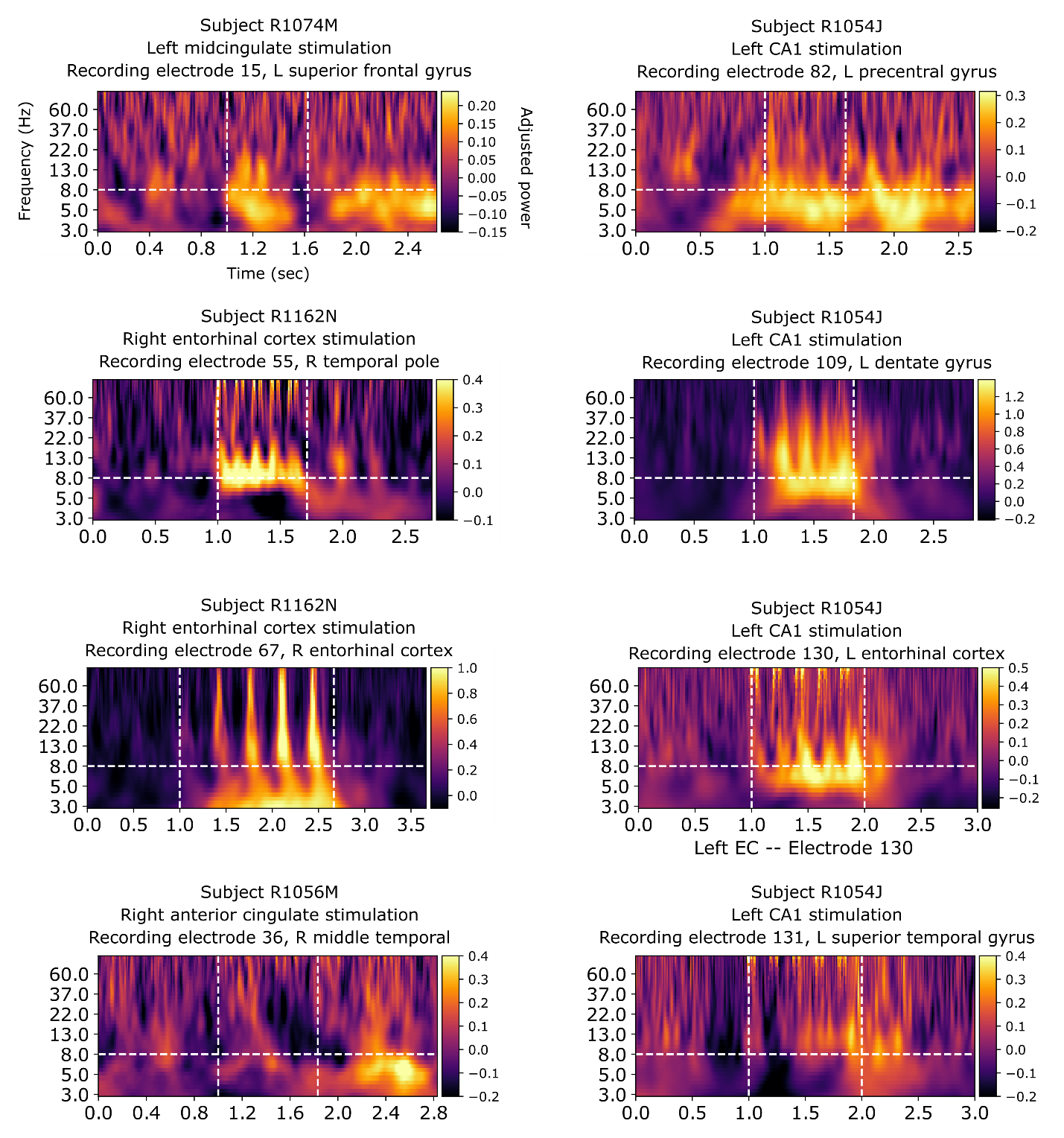


**Supplemental Figure 1. Example spectrograms of theta power response to TBS.** Structured as in Figure 4a.


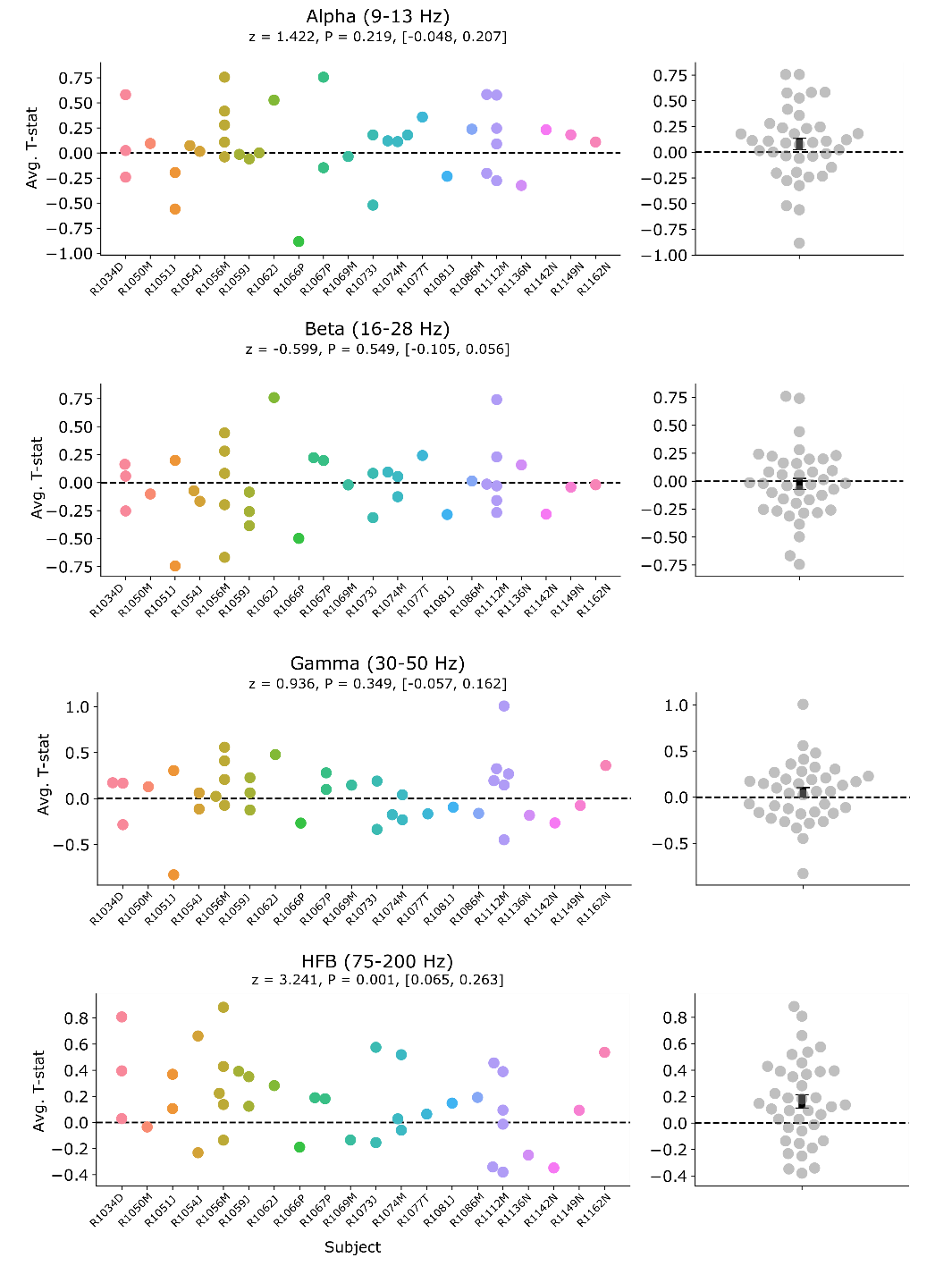


**Supplemental Figure 2. Stimulation-induced responses in higher frequency bands**. Structured as in Figure 2b-c. *t*-statistics are computed for each electrode representing the degree to which stimulation changes alpha (9-13 Hz), beta (16-28 Hz), gamma (30-50 Hz), or high-frequency broadband (HFB; 75-200 Hz) power. T-statistics are then averaged across all non-stimulated electrodes for each stimulation session/target. *Left:* The distribution of average *t*-statistics are shown for each subject, which are analyzed using a LMM to determine the population-level effect of stimulation. We found no significant response in any band except HFB (LMM Wald test, *z*=3.24, *P*=0.001, Intercept: [0.065, 0.263] 95% CI). *Right*: Distribution of average *t*-statistics across all sessions, error bars show +/- 1 SEM.


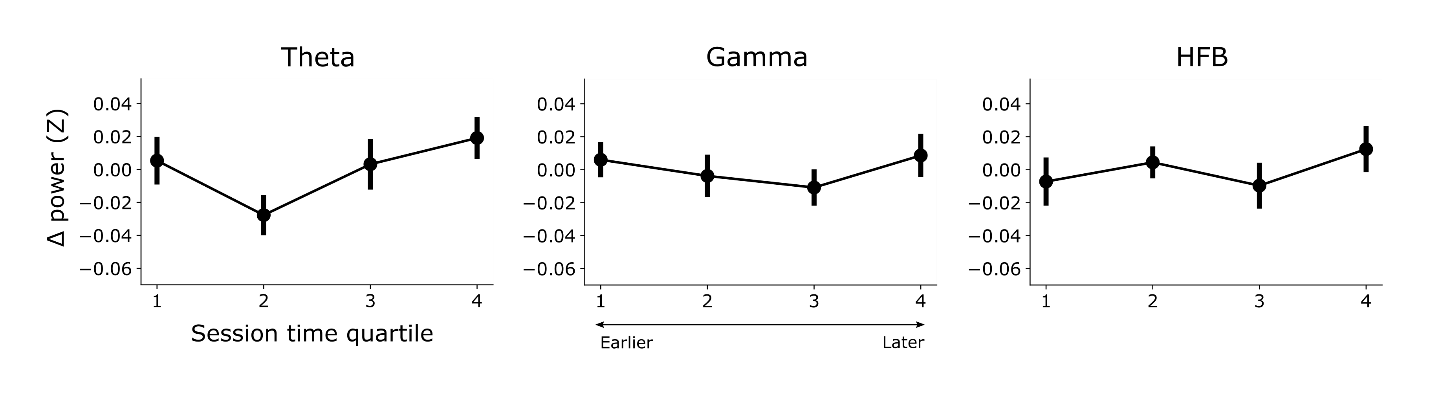


**Supplemental Figure 3.** Experimental sessions were binned into 6-minute quartiles in the theta-responsive subset of electrodes (N=29 sessions, 174 electrodes). The change (post-minus-pre) in spectral power induced by each stimulation event (Δpower) was z-scored across trials for each electrode, averaged over electrodes, and then averaged within each quartile. There was no significant effect of time on Δpower in any frequency band (repeated-measures ANOVA, *P* > 0.05). Error bars show +/- 1 SEM across sessions.

| Subject | Session | Stimulation Target | Stimulated electrode index | Number of responsive electrodes | Average brainwide theta t-statistic |
| --- | --- | --- | --- | --- | --- |
| R1034D | 2 | Left DG | 71 | 7 | 0.663284272 |
| R1050M | 0 | Left TC | 77 | 3 | 0.260830951 |
| R1051J | 0 | Left Precentral Gyrus | 27 | 1 | -0.117635098 |
| R1051J | 1 | Left Precentral Gyrus | 33 | 1 | 0.495637046 |
| R1142N | 0 | Left Middle Temporal Gyrus | 60 | 2 | 0.010018778 |
| R1054J | 0 | Left CA1 | 118 | 18 | 0.961617885 |
| R1054J | 1 | Left CA1 | 119 | 1 | -0.378963599 |
| R1056M | 0 | Right CA1 | 15 | 17 | 0.689995001 |
| R1056M | 1 | Right DG | 13 | 5 | 0.068599456 |
| R1056M | 2 | Right Supramarginal Gyrus | 92 | 4 | 0.282182057 |
| R1056M | 3 | Right Amy | 0 | 24 | 1.264917543 |
| R1056M | 4 | Right ACg | 55 | 13 | 0.492840737 |
| R1059J | 0 | Right EC | 91 | 15 | 0.461842255 |
| R1059J | 1 | Right EC | 86 | 4 | 0.530376995 |
| R1059J | 2 | Right EC | 81 | 1 | -0.047174213 |
| R1062J | 0 | Right Precentral Gyrus | 126 | 3 | 0.177968677 |
| R1067P | 0 | Left CA3 | 112 | 21 | 1.333422146 |
| R1067P | 1 | Left CA2 | 113 | 2 | 0.277677331 |
| R1069M | 0 | Left DLPFC | 31 | 1 | 0.227644859 |
| R1073J | 0 | Left PRC | 64 | 1 | 0.121792976 |
| R1073J | 1 | Left PRC | 65 | 1 | -0.409417027 |
| R1074M | 1 | Left MCg | 46 | 7 | 0.349567418 |
| R1074M | 2 | Left DLPFC | 44 | 1 | 0.278792339 |
| R1077T | 0 | Left Sub | 99 | 9 | 0.427334136 |
| R1112M | 3 | Right DG | 3 | 1 | -0.173287273 |
| R1112M | 4 | Right CA1 | 6 | 4 | 0.478420071 |
| R1112M | 5 | Right Amy | 2 | 2 | 0.266311364 |
| R1149N | 0 | Left Middle Temporal Gyrus | 25 | 1 | -0.192357706 |
| R1162N | 0 | Right EC | 52 | 4 | -0.002735454 |

**Supplemental Table 1. Subjects/targets with positive theta response to TBS.**

| Subject | Session | Stimulation Target | Stimulated electrode index | Number of responsive electrodes | Average brainwide theta T-statistic |
| --- | --- | --- | --- | --- | --- |
| R1051J | 0 | Left Precentral Gyrus | 27 | 2 | -0.11764 |
| R1142N | 0 | Left Middle Temporal Gyrus | 60 | 1 | 0.010019 |
| R1054J | 0 | Left CA1 | 118 | 2 | 0.961618 |
| R1054J | 1 | Left CA1 | 119 | 2 | -0.37896 |
| R1056M | 0 | Right CA1 | 15 | 1 | 0.689995 |
| R1056M | 1 | Right DG | 13 | 2 | 0.068599 |
| R1056M | 2 | Right Supramarginal Gyrus | 92 | 1 | 0.282182 |
| R1056M | 4 | Right ACg | 55 | 3 | 0.492841 |
| R1059J | 0 | Right EC | 91 | 3 | 0.461842 |
| R1059J | 2 | Right EC | 81 | 1 | -0.04717 |
| R1062J | 0 | Right Precentral Gyrus | 126 | 5 | 0.177969 |
| R1066P | 0 | Left Sub | 14 | 1 | -0.43518 |
| R1073J | 0 | Left PRC | 64 | 1 | 0.121793 |
| R1073J | 1 | Left PRC | 65 | 1 | -0.40942 |
| R1074M | 1 | Left MCg | 46 | 2 | 0.349567 |
| R1077T | 0 | Left Sub | 99 | 1 | 0.427334 |
| R1081J | 0 | Left ACg | 56 | 2 | -0.2464 |
| R1112M | 1 | Right Amy | 1 | 1 | 0.13 |
| R1112M | 2 | Right DG | 4 | 3 | -0.57315 |
| R1112M | 4 | Right CA1 | 6 | 1 | 0.47842 |
| R1136N | 0 | Left EC | 29 | 1 | -0.21689 |
| R1162N | 0 | Right EC | 52 | 3 | -0.00274 |

**Supplemental Table 2. Subjects/targets with negative theta response to TBS.**
